# The Impact of a Specialized Hernia Center and Standardized Practices on Surgical Outcomes in Hernia Surgery: A Systematic Review and Meta-Analysis

**DOI:** 10.3389/jaws.2024.13270

**Published:** 2024-07-22

**Authors:** Carlos Andre Balthazar da Silveira, Ana Caroline Dias Rasador, Diego L. Lima, Raquel Nogueira, Valberto Sanha, João P. G. Kasakewitch, Leandro T. Cavazzola, Prashanth Sreeramoju, Flavio Malcher

**Affiliations:** ^1^ Bahiana School of Medicine and Public Health, Salvador, Bahia, Brazil; ^2^ Department of Surgery, Montefiore Medical Center, The Bronx, NY, United States; ^3^ Department of Surgery, Federal University of Health Science of Porto Alegre, Porto Alegre, Brazil; ^4^ Department of Surgery, Beth Israel Deaconess Medical Center, Harvard Medical School, Boston, MA, United States; ^5^ Department of Surgery, Federal University of Rio Grande do Sul, Porto Alegre, Brazil; ^6^ Division of General Surgery, New York University Langone, New York, NY, United States

**Keywords:** hernia center, ventral hernia, inguinal hernia, incisional hernia, hernia specialist

## Abstract

**Aim:** Hernia registries report that guidelines are not always implemented by general surgeons and suggest that the success rate of this procedure is higher in hernia specialty centers. There are many definitions of hernia centers, but their objectives consist of improving healthcare by homogenizing the clinical practice. We performed a systematic review and meta-analysis to analyze hernia centers’ definitions and compare hernia centers with non-specialized centers.

**Material and Methods:** Cochrane Central, Scopus, Scielo, and PubMed were systematically searched for studies defining a hernia center or comparing hernia centers and non-specialized centers. Outcomes assessed were recurrence, surgical site events, hospital length of stay (LOS), and operative time. We performed subgroup analyses of hernia type. Statistical analysis was performed with R Studio.

**Results:** 3,260 studies were screened and 88 were thoroughly reviewed. Thirteen studies were included. Five studies defined a hernia center and eight studies, comprising 141,366 patients, compared a hernia center with a non-specialized center. Generally, the definitions were similar in decision-making and educational requirements but differed in structural aspects and the steps required for the certification. We found lower recurrence rates for hernia centers for both inguinal (1.08% versus 5.11%; RR 0.21; 95% CI 0.19 to 0.23; *p* < 0.001) and ventral hernia (3.2% vs. 8.9%; RR 0.425; 95% CI 0.28 to 0.64; *p* < 0.001). Hernia centers also presented lower surgical site infection for both ventral (4.3% vs. 11.9%; RR 0.435; 95% CI 0.21 to 0.90; *p* = 0.026) and inguinal (0.1% vs. 0.52%; RR 0.15; 95% CI 0.02 to 0.99; *p* = 0.49) repair.

**Conclusion:** Our systematic review and meta-analysis support that a hernia center establishment improves postoperative outcomes data.

**Systematic Review Registration:** https://www.crd.york.ac.uk/prospero/display_record.php?ID=CRD42024522263, PROSPERO CRD42024522263.

## Introduction

Over the last two decades, there has been an increase in the alternatives of hernia surgery for both open and minimally invasive procedures [1]. Considering the constant emerging evidence and technologies regarding abdominal wall surgery, with novel surgical devices and techniques, it has become harder and more demanding for the general surgeon to master the new advances and manage patients using a tailored approach [1, 2]. In this regard, there has been debate concerning the need for the accreditation and certification of hernia centers, aiming to establish guideline-based practices and provide education and specialization in hernia surgical techniques to improve the quality of hernia surgery [3–5].

Despite the hernia repair being a common procedure in the general surgeon’s routine, evidence suggests that the success rate of this procedure is lower when compared to a hernia specialist’s or a hernia specialty center’s rate [6]. Gilbert et al. [7] showed that general surgeons have a significantly higher recurrence incidence following hernia procedures. In addition, despite the scientific evidence brought by the most recent literature, general surgeons often do not follow the guidelines regarding the adoption of these new practices in their clinical approach [2, 7].

The certification process for hernia centers has been implemented by some societies and organizations worldwide, such as the German Hernia Society (GHS) along with the German Society of General and Visceral Surgery [8], and others have proposed accreditation requirements, such as the Italian Society of Hernia and Abdominal Wall Surgery [9]. Despite the definitions of hernia centers being different between the societies, their purposes consist of improving healthcare in hernia surgery by homogenizing the clinical practice and by following the guidelines in a standardized manner [10].

No previous systematic review and meta-analysis is available assessing the outcomes of hernia center facilities, therefore, we aimed to perform a systematic review and meta-analysis to compare hernia centers with non-specialized centers regarding intraoperative and postoperative outcomes.

## Methods

This meta-analysis was performed in accordance with the Preferred Reporting Items for Systematic Review and Meta-Analyses (PRISMA) Statement and recommendations from the Cochrane Collaboration Handbook for Systematic Reviews of Interventions [11]. We prospectively registered our research protocol in the International Prospective Register of Systematic Reviews (PROSPERO) (ID CRD42024522263)

### Eligibility Criteria

In the qualitative systematic review, we included all studies that defined a hernia center or presented data regarding hernia center outcomes/patient characteristics. For the meta-analysis, we included studies that met all the following eligibility criteria: 1) Defined a hernia center; 2) included patients undergoing ventral hernia repair (VHR) or inguinal hernia repair (IHR); 3) compared the hernia center sample with a control group of non-specialized center or pre-quality improvement/hernia center certification. We excluded studies with 1) analysis of experience instead of the definition of a hernia center, 2) no control groups, 3) conference abstracts, 4) editorials or 5) reviews.

### Search Strategy and Data Extraction

Two authors (C.S. and A.R.) independently and systematically searched PubMed, Embase, Cochrane Library, ScieLO (Scientific Electronic Library Online), and LILACS (Literatura Latino Americana em Ciencias da Saúde) from inception to 15 October 2023. The following terms were used without filters, publication date, or language restrictions: (“specialty” OR “referral centers” OR “reference units” OR “center of reference” OR “specialized surgeon” OR “specialized surgeons” OR “hernia center” OR “abdominal wall surgery center” OR “hernia specialty” OR “hernia centre” OR “hernia centers” OR “hernia centres” OR “abdominal wall surgery specialization” OR “hernia service” OR “hernia specialist” OR “hernia specialists” OR “referral center” OR “hernia referral center” OR “referral centre” OR “referral centres” OR “hernia program” OR “abdominal wall program” OR “hernia unit” OR “abdominal wall unit” OR “abdominal wall surgery unit” OR “dedicated hernia” OR “hernia dedicated”) AND (hernia OR abdominal wall). The references from all included studies, previous systematic reviews, and meta-analyses were also searched manually for any additional studies. Eventual conflicts were resolved by consensus among the authors. Two authors (C.S. and A.R.) independently extracted the following data from selected studies: 1) country, 2) number of patients, 3) study design, 4) hernia center definition, and 5) year.

### Quality Assessment

We evaluated the risk of bias using the Cochrane Risk of Bias Assessment Tool for Non-Randomized Studies (ROBINS-I) [12] for comparative studies, wherein each study is scored as high, moderate, or low risk of bias. The assessment was performed by two independent authors (J.K. and V.S.), and disagreements were resolved through consensus after discussing reasons for discrepancies.

### Outcomes

Data was analyzed separately for inguinal and ventral hernias. Our outcomes consisted of postoperative events, such as 1) recurrence, 2) surgical site infection (SSI), 3) seroma, 4) hematoma, 5) reoperation, and 6) mortality rates.

We also collected descriptive data regarding 1) hernia center definitions, 2) mesh use, 3) financial aspects, and 4) referral patterns before and after hernia centers’ establishment.

### Statistical Analysis

We computed risk ratios (RR) using the Mantel-Haenszel test for dichotomous outcomes and used 95% confidence intervals (CI) to measure effect size. We considered *p*-values of less than 0.05 to be statistically significant. We used mean differences (MD) as the effect measure for continuous outcomes, with 95% CI.

To assess heterogeneity, Cochran’s Q test and I^2^ statistics were utilized. We classified I^2^ values of <25%, 25%–75%, and >75% as representing low, moderate, and high heterogeneity, respectively. To account for potential disparities in both clinical and methodological aspects across studies, we applied the restricted maximum-likelihood estimator and random effects models for outcomes presenting with moderate to high heterogeneity. We performed sensitivity analyses using leave-one-out analysis for outcomes presenting statistically significant results with high heterogeneity. Publication bias was assessed for all the outcomes which included more than 10 studies by Egger’s Test. Furthermore, we performed a funnel plot to investigate heterogeneity between study-specific estimates. Our meta-analysis used the metafor package for RStudio version 4.2.2 (R Foundation for Statistical Computing, Vienna, Austria).

## Results

### Study Selection and Characteristics

**FIGURE 1:**
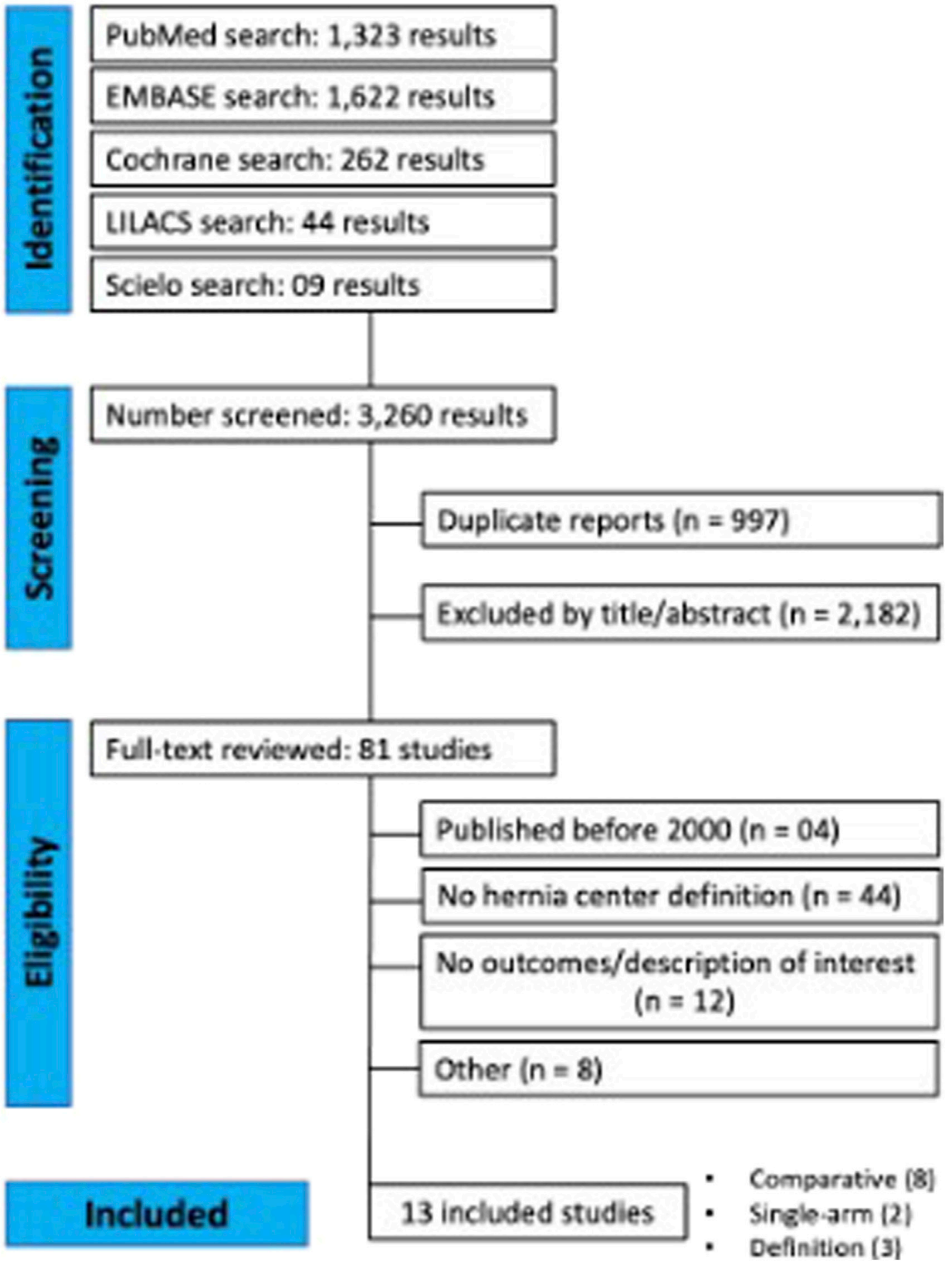
PRISMA flowchart of selected studies.

The initial search yielded 3,260 results. After removing duplicate studies, 2,263 records were identified through database searching, and their summaries were screened for eligibility. Of these, 81 remained and were fully reviewed based on predefined eligibility criteria. A total of 8 comparative studies were included, comprising 141,366 patients, of whom 81,989 (58%) were in the hernia center group (Figure 1). Two single-arm studies were also included for quantitative analysis. The studies’ characteristics are detailed in Table 1.

**TABLE 1:** Characteristics of the studies included.

Author	Study type	Year	Hernia
Cheong et al [13]	Single-arm retrospective cohort	2014	Inguinal
Cherla et al [14]	Comparative retrospective cohort	2017	VHR
Haskins et al [4]	Database (ACHQC) comparative retrospective cohort	2023	VHR
Katzen et al [15]	Comparative retrospective cohort	2023	VHR
Krpata et al [5]	Comparative retrospective cohort	2016	All
Malik et al [16]	Comparative retrospective cohort	2016	Inguinal
Pereira et al [17]	Comparative retrospective cohort	2019	VHR
Rodrigues-Gonçalves et al [18]	Comparative retrospective cohort	2023	Inguinal
Willms et al [6]	Comparative retrospective cohort	2023	VHR + Inguinal
Williams et al [19]	Single-arm retrospective cohort	2014	All hernias

VHR, Ventral hernia repair; ACHQC, abdominal core health quality collaborative.

### Quality Assessment

**FIGURE 2:**
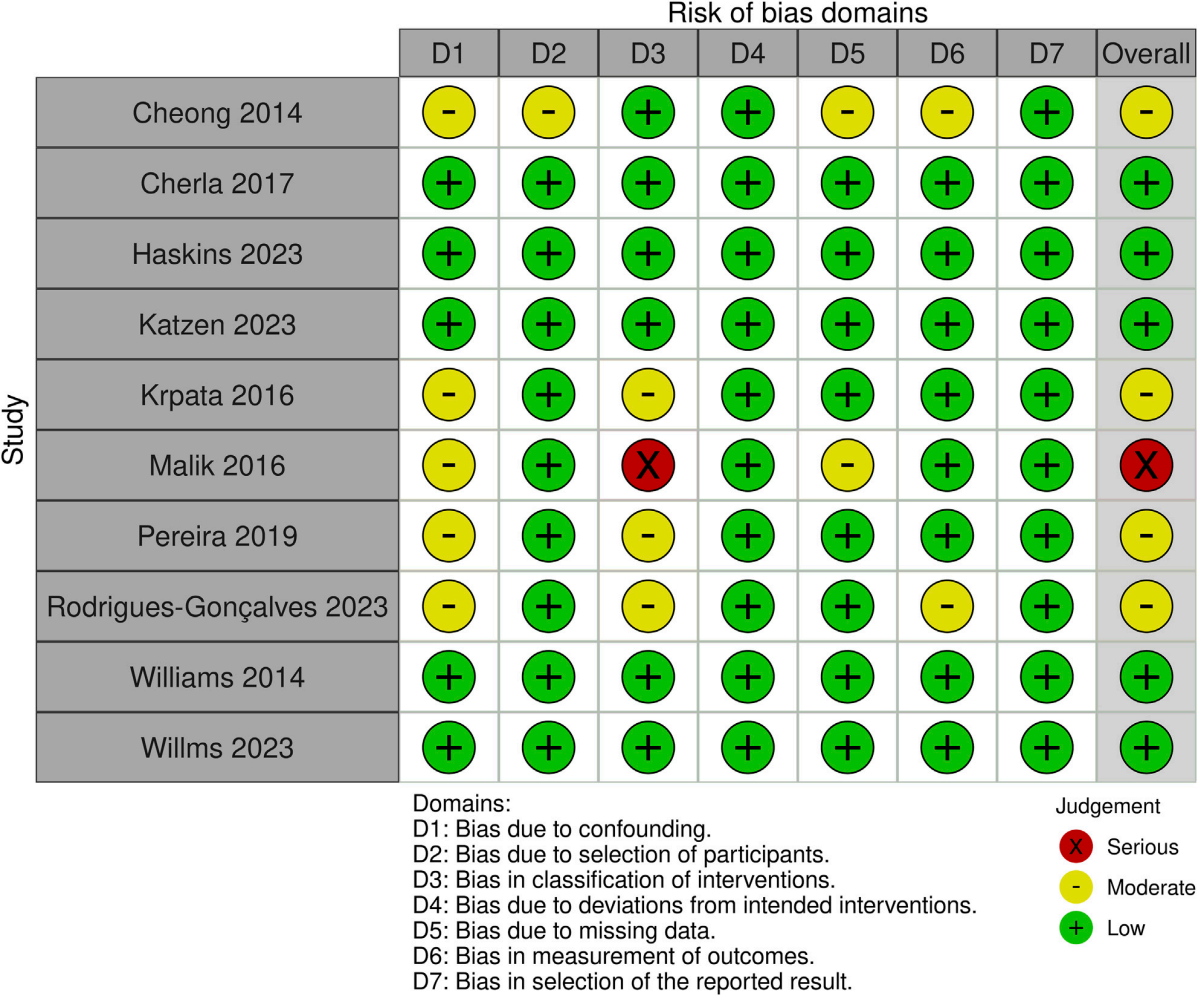
Risk of bias of included studies.

We used the ROBINS-I tool in the risk of bias analyses for all the included studies. Five studies were rated as low risk of bias, four as having a moderate risk of bias, and one as with a serious risk of bias. Overall reasons for the risk of bias between the moderate to serious risk studies raised from confounding factors, selection of participants, classification of interventions, missing data, or measurement of outcomes. Full risk of bias analyses and specific domain rating of individual studies are presented in Figure 2.

### Hernia Center Definition

We included three studies comprising hernia centers’ definitions, including technical, structural, educational, and scientific requirements for a hernia center certification according to the European Hernia Society (EHS) [10], the German Hernia Society [8], and the Italian Hernia Society [9]. Almost all societies divide the process into steps of specialization until the unit reaches the classification of reference or highly specialized center. Both the Italian and German Hernia Society divide the specialization into three steps, while the EHS society used non-specific divisions. The most common requirements for all three societies are focused on the number of specialists available, structural aspects, including intensive care unit, outpatient clinic, and material for minimally invasive surgery, and also updated with training, including attending scientific meetings yearly. Full specialization requirements are available in Table 2.

**TABLE 2:** Hernia center definitions.

Study	Steps	Specialized surgeons	Structural aspects	Decision-making	Education and science	Data management
ACCESS Project, 2019 (Europe—EHS)	Certification levels and requirements to upgrade to a high level	Experienced surgeons meeting annual caseload and conference requirements	QI conferences; diagnostic tools (CT, MRI); MIS equipment ICU.	Current scientific recommendations	Staff responsible for science, education, and training programs	Cases register prospectively in a registry or quality database
Stabilini, 2018 (Italy)	(A)First level (Single surgeon)	(A)General surgeon + Minimum learning curve for all procedures and minimum year caseload	—	Current scientific recommendations	—	—
	(B) Referral centers (at least 2 surgeons)	(B) Minimum 1 year after “A” + Members of the society, with a minimum year caseload + Plastic surgeon available	(B) Outpatient clinic Emergency service ICU Transfusion center Diagnostic (CT, Laboratory) Advanced wound management	Current scientific recommendations	(B)Training site for the Italian School + Provide data + Attend to 3 meetings/workshops yearly + EHS meeting each 2 years	—
	(C) Highly specialized (at least 2 board surgeons and a fellow surgeon)	(C) Minimum 1 year after “B” + Formal research assigned surgeon fellow, PhD or resident	(C) Same as “B”	Current scientific recommendations	(C) Yearly: Organize 1 course + 2 of the following: 1 publication or collaborative trial organization or EHS meeting participation or research on new technologies	—
Köckerling, 2014 (Germany)	(A) Seal of participation in a society-registered database (3 years minimum)	(A) Surgeons must be full members of the German and European Hernia Societies	—	Current scientific recommendations	—	(A) Cases registered in the Herniamed Registry (60% follow-up data in 3 years)
	(B) Competence center (minimum 1 year of the seal of participation)	(B) “A” requirements + At least 1 meeting/conference yearly	(B) Monthly QI conferences; Special consultations weekly for the patients; Postoperative pain regimen protocol	Current scientific recommendations	—	(B) All “A” requirements + 1-year follow-up for 60% of the patients
	(C) Reference center (minimum 2 years of competence center)	(C)All “B” requirements + Plastic surgeon available	(C) All “B” requirements + Facilities to perform all laparoscopic procedures	Current scientific recommendations	(C) Education seminars and guest visits credited by medical board + 2 publications or presentations at meetings	(C) All the previous requirements

EHS, European hernia society; QI, quality improvement; CT, computerized tomography; MRI, magnetic resonance imaging; MIS, minimally invasive surgery; ICU, intensive care unit.

Furthermore, there are specific criteria used for hernia center definition by the German and Italian Hernia Society which were based on the center’s annual caseload and a maximum complication rates cutline. In this regard, for the German Hernia Society, the unit needs to present a total of 250 hernia repairs per year, comprising at least 50 incisional hernia repairs, 5 complex hernias, and 5 hiatal hernias. On the other hand, the Italian Society requires a minimum of 150 inguinal hernia repairs, comprising 30 complex cases, and a minimum of 50 abdominal wall reconstructions, comprising 20 complex cases yearly. Concerning postoperative complication rates, both societies analyzed maximum surgical site infection rates depending on hernia type and surgical approach. Also, there were specific criteria used by each society regarding recurrence and other postoperative complication rates, such as chronic pain and mortality. Full postoperative complication and annual caseload requirements are available in Table 3.

**TABLE 3:** Minimum procedural volume and maximum complication rates requirements for a hernia center.

Study	Steps	Learning curve	Minimum volume (per year)	Maximum complication rates					
				Recurrence (1 year)	Reoperation	Mortality	General complications (%)	Infection (%)	Chronic pain
Köckerling, 2014 (Germany)	(A) Seal of participation in a society-registered database	—	30 hernia patients/year	—	Inguinal: <2% Incisional: <10%	—	Inguinal: <5	Open incisional: <10 Laparoscopic Incisional: <3	—
	(B) Competence center	—	200 hernia operations/year (30 incisional)						
	(C)Reference center	—	250 hernia operations/year (50 incisional; 5 complex; 5 hiatal)						
Stabilini, 2018 (Italy)	(A) First level (Single surgeon)	120 Inguinal Hernias (60 MIS and 60 open) 40 AWR (20 MIS and 20 open)	50 Inguinal Hernias (25 MIS and 25 open) 50 AWR (25 MIS and 25 open)	Inguinal: <2% AWR: <5% Complex AWR: <10%	—	Inguinal: <0.5% AWR: <1% Complex AWR: <5%	Inguinal: <10 AWR: <30 Complex AWR: <50	Inguinal: <3 AWR: <10 Complex AWR: <30	Inguinal: <15%
	(B) Referral centers	—	100 Inguinal Hernia 50 AWR (10 complex cases)						
	(C) Highly specialized centers	—	150 Inguinal Hernia (20 complex cases) 50 AWR (20 complex cases)						

MIS, Minimally invasive surgery; AWR, abdominal wall reconstruction.

Among the comparative studies included, two analyzed IHR only, four studies analyzed VHR, one analyzed both IHR and VHR separately, and one analyzed both IHR and VHR together.

### Ventral Hernia Repair

**FIGURE 3:**
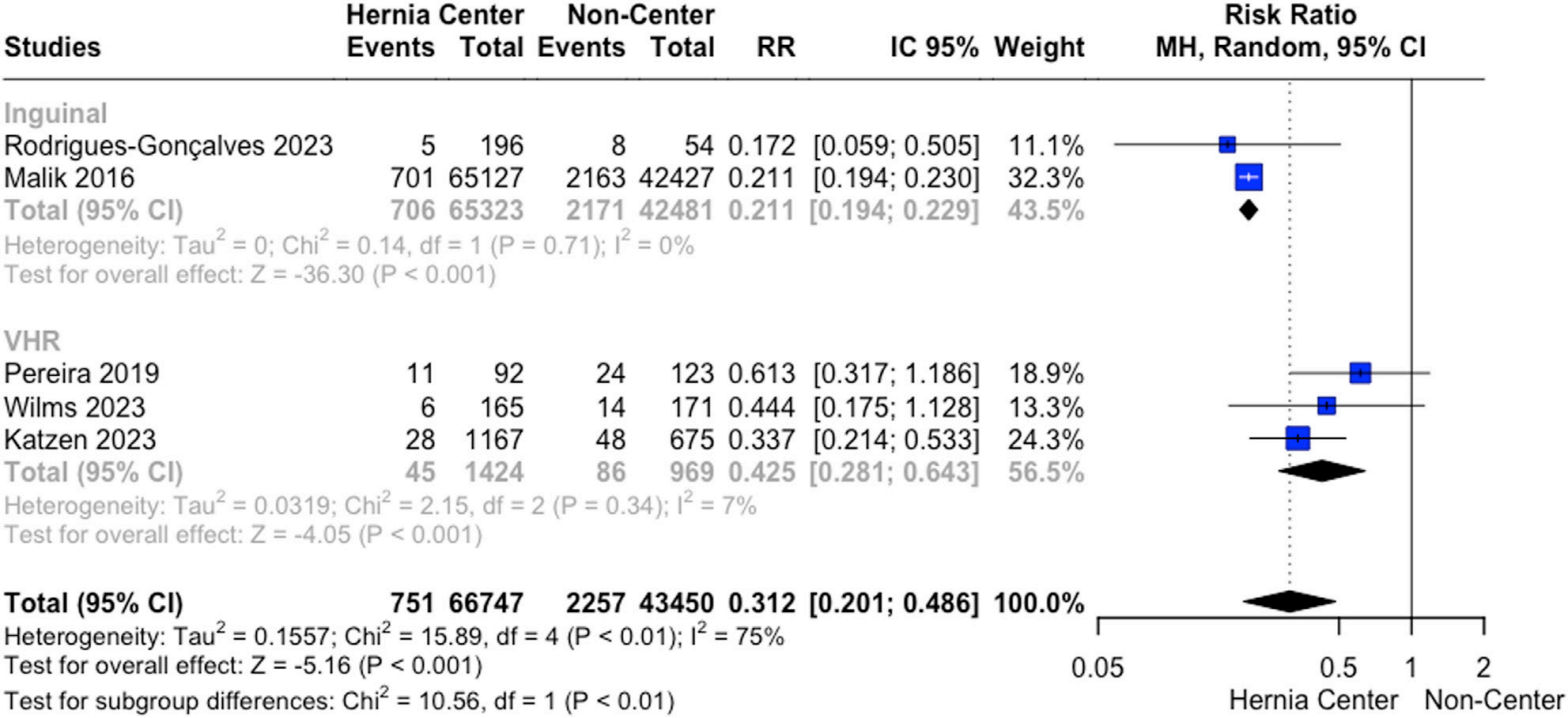
Recurrence rates following VHR and IHR.

Three studies analyzed recurrence rates for VHR. We found lower recurrence rates for surgeries performed in hernia centers (3.2% vs. 8.9%; RR 0.425; 95% CI 0.28 to 0.64; *p* < 0.001; I^2^ = 7%; Figure 3). Leave-one-out sensitivity analysis showed no differences in heterogeneity reduction or loss of significance. However, no statistically significant differences were found in reoperation rates between the groups in the analysis with three studies (1.3% vs. 1.4%; RR 0.68; 95% CI 0.33 to 1.40; *p* = 0.3; I^2^ = 47%).

**FIGURE 4:**
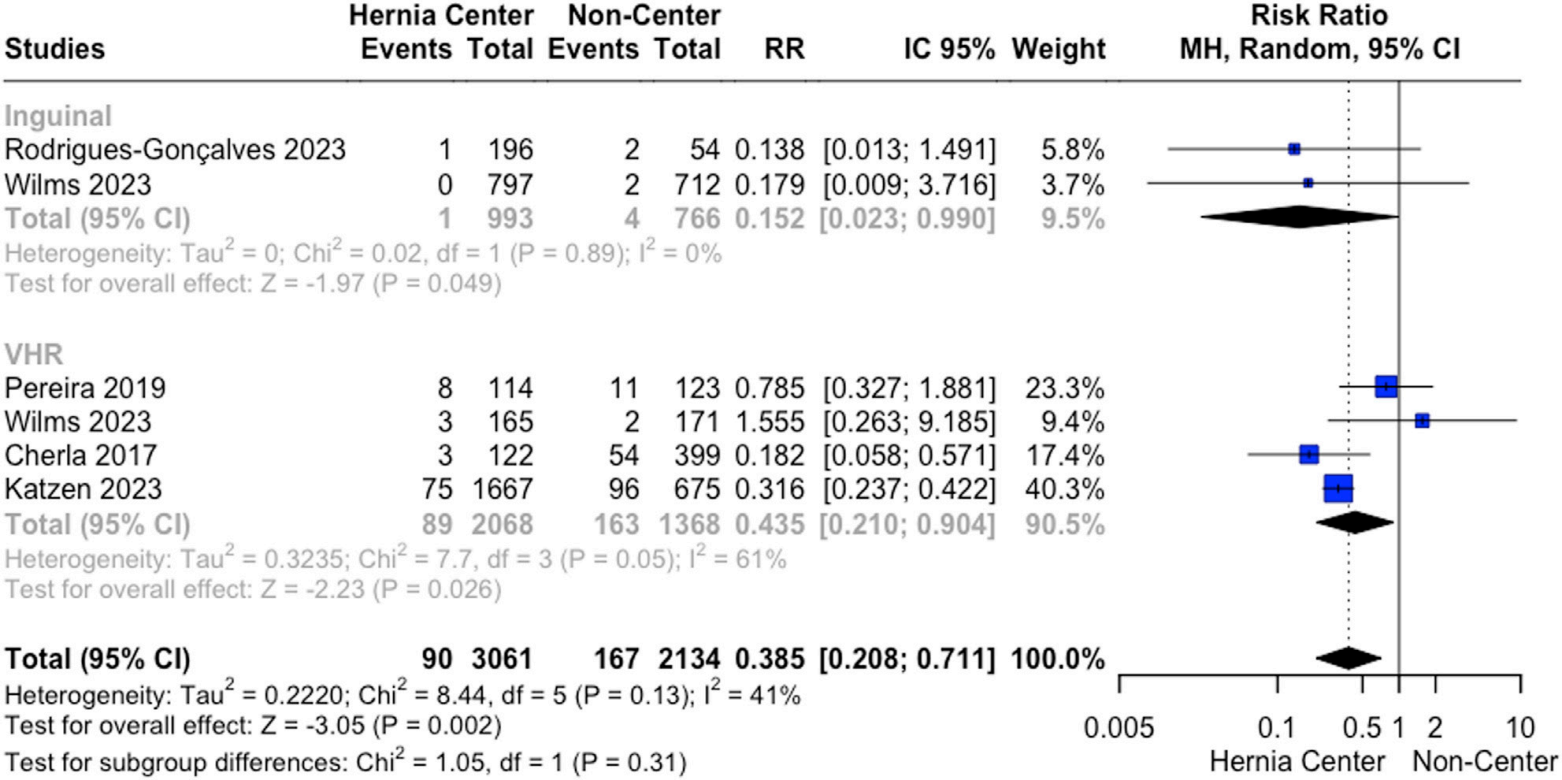
A. Surgical site infection rates following VHR and IHR.

SSI following VHR was analyzed by four studies. Specialized hernia centers presented a lower SSI rate following VHR (4.3% vs. 11.9%; RR 0.435; 95% CI 0.21 to 0.90; *p* = 0.026; I^2^ = 61%; Figure 4). Leave-one-out sensitivity analysis showed no differences in heterogeneity reduction or loss of significance.

We analyzed seroma rates with three available included studies. No statistically significant differences were found between the groups (9% vs. 10.9%; RR 0.81; 95% CI 0.64 to 1.04; *p* = 0.098; I^2^ = 0%). Also, we found no differences between the groups in hematoma rates (0.79% vs. 0.95%; RR 0.53; 95% CI 0.16 to 1.68; *p* = 0.29). The statistical significance did not change after the leave-one-out sensitivity analyses of both seroma and hematoma rates.

**FIGURE 5:**
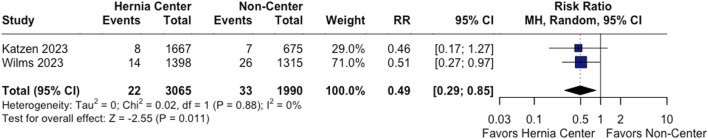
Mortality rates following VHR.

Only two studies analyzed mortality rates. We found a reduction in mortality for specialized hernia centers (0.72% vs. 1.66%; RR 0.49; 95% CI 0.29 to 0.85; *p* = 0.01; I^2^ = 0%; Figure 5).

### Inguinal Hernia Repair

Recurrence rates after IHR were analyzed by two studies. The pooled analysis showed a lower recurrence for hernia centers (1.08% vs. 5.11%; RR 0.21; 95% CI 0.19 to 0.23; *p* < 0.001; I^2^ = 0%; Figure 3). Also, we found that the effect of hernia centers on recurrence reduction for IHR was even more impactful compared to VHR (Test for subgroup differences *p* < 0.01; Figure 3).

Two studies analyzed SSI rates for IHR. Our analysis showed a reduction in SSI for specialized hernia centers (0.1% vs. 0.52%; RR 0.15; 95% CI 0.02 to 0.99; *p* = 0.49; I^2^ = 0%; Figure 4).

**FIGURE 6:**
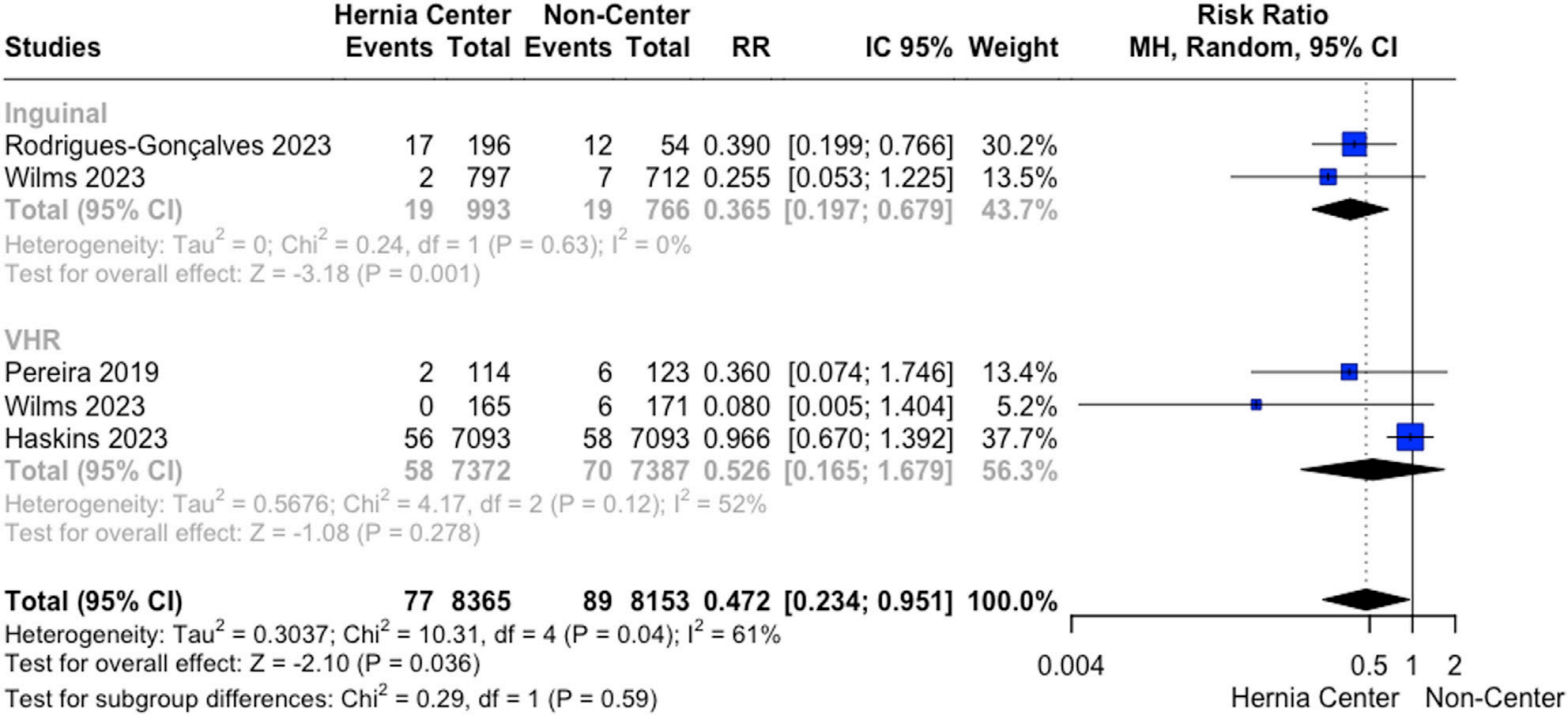
Hematoma rates following VHR and IHR.

Also, we found a significant reduction in hematoma rates for specialized centers (RR 0.365; 95% CI 0.2 to 0.68; *p* = 0.001; I^2^ = 0%; Figure 6). No statistically significant difference was found in seroma rates between the groups (RR 0.367; 95% CI 0.034 to 3.965; *p* = 0.41; I^2^ = 84%).

## Discussion

In this comprehensive systematic review and meta-analysis comprising 141,366 patients, we found that specialized hernia centers were associated with a lower recurrence and lower SSI for both IHR and VHR. Also, specialized centers presented lower mortality rates for VHR and a reduced hematoma incidence for IHR. No differences were seen regarding seroma and reoperation rates for both IHR and VHR.

The first centers dedicated to hernia surgery emerged in the 1980s, with a common focus on standardizing surgical techniques for better patient treatment [20]. Since then, despite the interest in establishing specialized centers, societies criticized hospitals for self-claiming specialized hernia centers without specific criteria for such a definition [8]. In 2014, aiming to address this issue, the German Hernia Society proposed the creation of a pathway for considering a center as specialized in hernias, focusing on postoperative outcomes [8]. Our review highlighted the status of the literature regarding definitions of what constitutes a specialized hernia center. Despite divergences among societies, there are common features among them, focusing on prospective data registration, regular participation in annual meetings, availability of advanced technology encompassing the latest hernia surgical techniques and support for complications, as well as a minimum annual caseload, and most importantly, expected annual complication rates. In terms of the complexity of annually operated cases, societies recommend that 10%–20% of the total annual ventral hernias operated be considered complex hernias [8, 9], according to the complexity definition proposed by Slater et al [21]. In this sense, the hernia center should serve as a reference center, where patients with complicated conditions have optimal access to technology and skilled surgeons for their care [22, 23].

To achieve those results, establishing cutoffs for outcomes such as recurrence rates becomes necessary. Our pooled analysis found reduced recurrence rates for specialized centers, with 3.2% and 1.08%, compared to 8.9% and 5.11% recurrence incidence for non-specialized centers, for VHR and IHR, respectively. This is an important quality marker to support the complications cutoff establishment for hernia center definition. The Italian society recommends a recurrence rate of less than 2% for inguinal, 5% in 1 year, and less than 15% in 3 years for ventral hernia repair, which is consistent with our findings [9]. Furthermore, in their definition, it is described specific cutoffs for complex cases. This definition is crucial as it encompasses the previous requirement of the minimum annual caseload of complex surgeries, being the complications cutoff grounded in the contemporary literature on complications associated with complex and non-complex cases separately. That definition is particularly vital post-establishment of the unit as a reference center. Individual studies have shown that the admitted patient profile becomes more complex with the establishment of a specialized center, attracting patients from greater distances [22, 23], making it imperative to stratify the expected complication rates based on the complexity of each case.

Parallel to recurrence rates, the hernia center’s SSI cutoffs are also important postoperative quality markers, as increased infection rates are directly related to recurrence, increased length of hospital stay, and overall morbidity, especially in complex cases [14, 24, 25]. We found an increased SSI for non-specialized centers, while the pooled analysis of specialized hernia centers showed rates of 4.3% and 0.1% of SSI for VHR and IHR, respectively. Between the hernia center pathways guidelines, established cutoffs for SSI only for ventral hernia repair, which are defined as less than 10% as a consensus, and as less than 30% for complex cases [8, 9]. In addition, it is expected that a specialized hernia center presents less than 1% of mortality rates for VHR. We found a mortality rate of 0.72% for hernia centers, compared to 1.66% for non-specialized centers, also supporting guidelines recommendations.

Furthermore, it is important to highlight that the process of becoming a specialized hernia center needs to be based on steps. In addition to societies, individual studies propose pathways for establishing a specialized hernia surgery center. Smith et al [3], in this context, dissect the structure and distribution of a hernia-based service, suggesting that it should involve the reception of referred patients, with clinical and imaging evaluation, proper preoperative optimization, as well as adequate follow-up for necessary patient feedback. Current evidence suggests that this longitudinal process, including preoperative adequate management, reduces postoperative complications, and may be a part of the hernia center establishment criteria [26–29]. Despite adjustments for baseline comorbidities, hernia complexity, and intraoperative complications, our findings of reduced complications for hernia centers may be justified by evidence-based decision-making, including the listed preoperative optimization and choice of adequate surgical techniques by trained surgeons. However, a national profile study conducted by Shulkin et al showed that among hernia center surgeons, only 3.3% are hernia board certified [23]. This finding highlights the importance of not only the academic title but also the experience and annual caseload of the surgeons as an expertise parameter.

Finally, it is important to highlight our analysis limitations. First, it is important to highlight that all hernia center literature was written by authors from hernia centers, which can generate bias associated with the complexity of the cases operated on, as well as the technical capability of these surgeons. Also, the definitions of hernia center were very heterogeneous between the studies. However, we tried to control and share specific heterogeneity by providing Cochran’s Q test and I^2^ statistics for each outcome analyzed. Furthermore, almost all included studies did not present separate data according to the surgical technique and approach (minimally invasive or open), so a subgroup analysis of those groups was not available. However, we believe that the results found on overall analyses would be similar for individual technique and surgical approach results, demonstrating hernia center’s fewer complications compared to non-specialized centers. Also, our qualitative analysis evidenced some studies as presenting a moderate and serious risk of bias, which also limits our data extrapolation. However, we made a comprehensive analysis including all clinical studies available on the topic, providing the only pooled analysis on this topic in the current literature.

## Conclusion

Our systematic review and meta-analysis support that hernia center establishment improves postoperative outcomes data for both inguinal and ventral hernia repair. We found lower recurrence, SSI, and hematoma rates for hernia centers compared to non-specialized centers. These findings highlight the potential of standardized and guideline-based interventions to improve patient outcomes and justify their consideration as an aim of future hernia societies’ discussions and establishment.
